# Localized *pmrB* hypermutation drives the evolution of colistin heteroresistance

**DOI:** 10.1016/j.celrep.2022.110929

**Published:** 2022-06-07

**Authors:** Natalia Kapel, Julio Diaz Caballero, R. Craig MacLean

**Affiliations:** 1 University of Oxford, Department of Zoology, 11a Mansfield Road, Oxford OX1 3SZ, UK

**Keywords:** antibiotic resistance, colistin, experimental evolution, *Pseudomonas aeruginosa*, heteroresistance, AMR, evolutionary rescue, mutation rate, hypermutability

## Abstract

Colistin has emerged as an important last line of defense for the treatment of infections caused by antibiotic-resistant gram-negative pathogens, but colistin resistance remains poorly understood. Here, we investigate the responses of ≈1,000 populations of a multi-drug-resistant (MDR) strain of *P*. *aeruginosa* to a high dose of colistin. Colistin exposure causes rapid cell death, but some populations eventually recover due to the growth of sub-populations of heteroresistant cells. Heteroresistance is unstable, and resistance is rapidly lost under culture in colistin-free medium. The evolution of heteroresistance is primarily driven by selection for heteroresistance at two hotspot sites in the PmrAB regulatory system. Localized hypermutation of *pmrB* generates colistin resistance at 10^3^–10^4^ times the background resistance mutation rate (≈2 × 10^-5^ per cell division). PmrAB provides resistance to antimicrobial peptides that are involved in host immunity, suggesting that this pathogen may have evolved a highly mutable *pmrB* as an adaptation to host immunity.

## Highlights

• *Pseudomonas* populations recover from colistin due to the growth of heteroresistant cells
• Heteroresistance is driven by pre-existing mutations in the PmrAB regulatory system
• *pmrB* mutations arise at 10^3^–10^4^ times the background mutation rate
• Heteroresistance is unstable and is rapidly lost in the absence of colistin

## Graphical abstract

**Figure:**
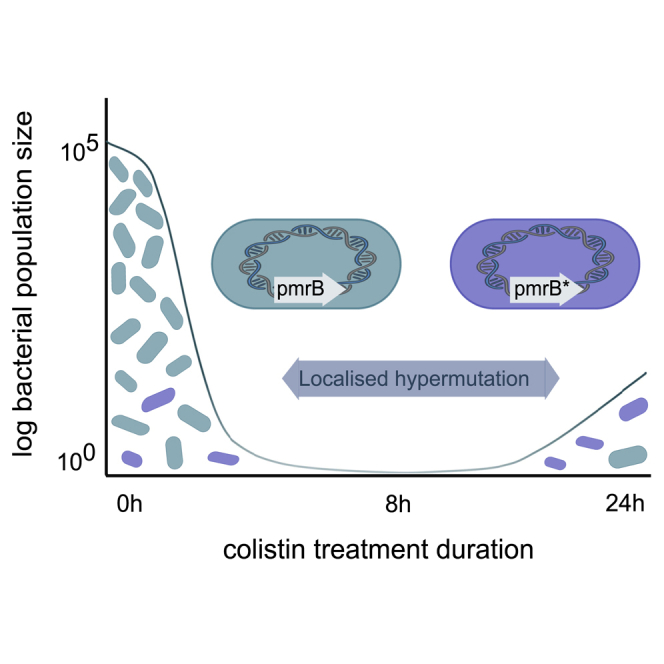

## Teaser

Colistin is important for the treatment of infections caused by antibiotic-resistant pathogens, but colistin resistance is poorly understood. Kapel et al. demonstrate that pre-existing mutations in the PmrAB regulatory system allow *P*. *aeruginosa* populations to rapidly adapt to colistin. Crucially, *pmrB* mutations occur at 10^3^–10^4^ times the background mutation rate.

## Introduction

The antimicrobial peptide colistin has emerged as an important last line of defense for the treatment of infections caused by multi-drug resistant (MDR) gram-negative pathogens, including *Escherichia coli*, *Klebsiella pneumoniae*, *Pseudomonas aeruginosa*, and *Acinetobacter baumanii* (Li et al., 2006; Michalopoulos et al., 2005; Reina et al., 2005). Given this, there is a pressing need to understand how bacterial pathogens adapt to colistin treatment.

Unfortunately, colistin has moderate efficacy for curing *P*. *aeruginosa* infections, although genetically elevated colistin resistance, as defined by clinical breakpoint minimum inhibitory concentrations (MICs), remains rare (Durakovic et al., 2011; Levin et al., 1999; Michalopoulos et al., 2005; Montero et al., 2009). One possible explanation for this is that poor pharmacodynamics prevent colistin from achieving high enough concentrations at the sites of *P*. *aeruginosa* infection (Li et al., 2006; Michalopoulos and Falagas, 2011). It is also possible that bacterial responses to colistin contribute to treatment failure. For example, treatment failure could be driven by adaptive changes in gene expression that confer colistin resistance *in vivo* (McPhee et al., 2003), by the growth of persister cells that survive colistin treatment (Balaban et al., 2019), or by heteroresister cells that carry unstable resistance mutations (Andersson et al., 2019). Importantly, in all these cases, we would not expect to see any stable increase in the resistance of isolates recovered after antibiotic treatment, as standardized methods for resistance testing rely on pre-culturing bacterial isolates in antibiotic-free medium.

The classical model for the evolution of resistance during infection is that antibiotic treatment selects for rare *de novo* resistance mutations that confer a stable antibiotic-resistant phenotype that is associated with fitness costs (Hughes and Andersson, 2017; MacLean et al., 2010; zur Wiesch et al., 2011). Given that resistance mutations occur at a low rate, this model is most applicable to cases where infection results in a large bacterial population or when bacterial mutation rates are high. Large populations of *P*. *aeruginosa* adapt to high concentrations of colistin in the lab through the sequential fixation of a series of mutations, generating elevated resistance (Dosselmann et al., 2017; Jochumsen et al., 2016). While this approach offers a powerful and elegant tool for understanding evolutionary trajectories and constraints, the clinical relevance of these experiments is unclear given that colistin resistance is rare in clinical settings.

Our approach to study responses to colistin was to expose ≈1,000 small populations (10^4^–10^5^ cells) of *P*. *aeruginosa* EP67, a colistin-sensitive MDR isolate (Wheatley et al., 2021), to the clinical breakpoint dose of colistin (2 mg/L). In this selective regime, populations could only persist over the long term if they were able to acquire increased antibiotic resistance. Our reasoning for this approach was that using many small populations of bacteria prevents rare mutations (i.e., 10^-8^–10^-9^ per cell division [Andersson and Hughes, 2009; Krasovec et al., 2017]) that confer high-level resistance from dominating the evolutionary response to antibiotic treatment (Vogwill et al., 2016). A further advantage of this approach was that studying responses to antibiotic treatment in small populations opened up the possibility that bacterial populations were driven to extinction before resistance could evolve (Gifford et al., 2018; Papkou et al., 2020), which is the most common outcome of antibiotic treatment in the clinic (Fish et al., 1995; Levin et al., 1999; Michalopoulos et al., 2005; Montero et al., 2009).

## Results and discussion

### Population responses to colistin treatment

To investigate the response of *Pseudomonas* populations to colistin, we inoculated 100 independent replicate cultures of EP67 into fresh culture medium containing the clinical breakpoint concentration of colistin (2 mg/L). Given that colistin-resistance mutations are rare in clinical isolates, we carried out this experiment using small populations (≈10^5^ colony forming units [CFUs]) that were very unlikely to contain any “classical” resistance mutations, which typically occur at a rate of 10^-8^–10^-9^ per cell division (Andersson and Hughes, 2009; Krasovec et al., 2017).

**Figure 1:**
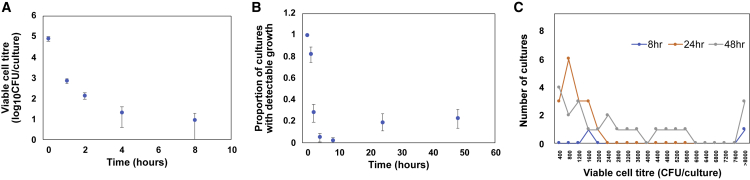
Population responses to colistin treatment Independent cultures of strain EP67 (n = 100) were inoculated into culture medium supplemented with colistin (2 mg/L). (A) Viable cell titer (n = 100; ±95% confidence interval [CI]) over the first 8 h of incubation. (B) Proportion of cultures (n = 100; ±95% CI) showing detectable growth over time, with a minimal limit of detection of 400 CFUs/culture. (C) Distribution of viable cell titer in cultures showing growth at 8, 24, and 48 h. The limits of detection in this assay were 400–8000 CFUs.

Viable cell density rapidly declined, and after 8 h of incubation, we recovered viable colonies from only 2/100 populations, giving an estimated cell density of only 10 CFUs/culture (Figures 1A and 1B). The number of populations that gave detectable growth increased to 15%–20% after continued incubation in colistin-containing medium, demonstrating that a small subset of EP67 populations eventually recovered from colistin treatment. The viable cell density in populations showing evidence of recovery increased between 24 and 48 h (rank-sum test, Z = 1.99, p = 0.046) but remained very low, with densities typically <5,000 CFUs (Figure 1C). This pattern of population crash and then recovery suggests that population recoveries were driven by the growth of a sub-set of cells with a colistin-resistant phenotype.

The low frequency of population recovery after colistin treatment suggests that population recovery was driven by rare events, such as spontaneous mutation or gene amplification. To better understand the frequency and repeatability of population recovery, we repeated this assay 6 times, with a total of 933 independent cultures. In these “population recovery assays,” we focused only on measuring the density of viable cells after 24 and 48 h of incubation to quantify the frequency of population recovery under colistin treatment. Population recovery was detected in all assays, but the frequency of recovery differed quite widely between assays (Table 1).

**Table 1:** Summary of population-rebounding experiments

Experiment	Number of replicate cultures	Initial CFUs/culture	Number of viable cultures	
			24 h	48 h
Population dynamics (Figure 1)	100	7.09 × 10^4^	19	23
Recovery 1	171	5.62 × 10^3^	6	9
Recovery 2	162	1.67 × 10^4^	64	52
Recovery 3	60	7.14 × 10^3^	2	32
Recovery 4	180	6.71 × 10^3^	54	99
Recovery 5	180	9.20 × 10^3^	37	75
Recovery 6	270	8.68 × 10^3^	13	17

This table shows a summary of the frequency of *Pseudomonas* population recovery in replicate colistin challenge experiments.

### Assessing the stability of evolved colistin resistance

**Figure 2:**
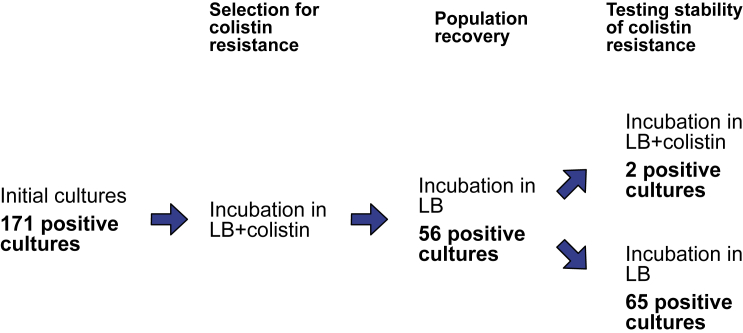
Testing the stability of colistin resistance Independent cultures of EP67 were incubated with colistin for 24 h, to select for resistance (n = 171). Cultures were then transferred to LB to allow resistant populations to expand. Recovered populations were then passaged into medium with LB or LB + colistin to test the stability of colistin resistance. After each passage, cultures diluted 10-fold and spotted out on LB agar plates to determine population viability, as determined by confluent colony growth.

Population recovery in the presence of colistin could have been driven by the growth of either classical resistant mutants (Dosselmann et al., 2017; Jochumsen et al., 2016) or heteroresistant cells (Nicoloff et al., 2019). To discriminate between these possibilities, we carried out a further experiment to measure the stability of colistin resistance under the assumption that resistance mutations are stable, whereas heteroresistance is intrinsically unstable (Figure 2). In this “stability” experiment, we first selected for colistin resistance, as in our previous experiments. Populations that were selected for colistin resistance were then passaged into colistin-free culture medium to allow populations that survived treatment to expand. These recovered populations were then passaged into medium containing colistin to measure the stability of the colistin-resistant phenotype and colistin-free medium as a control. Strikingly, colistin resistance was only maintained in 2 of the 56 populations (3.5%) that recovered from initial colistin treatment, allowing us to rule out the possibility that population recovery was driven by classical resistance mutations.

### Hypermutability of pmrB drives heteroresistance

The instability of colistin resistance suggests that the evolutionary response to colistin treatment was driven by selection for unstable genetic changes (i.e., gene amplification, and SNPs) that generate heteroresistance (HR) in *Pseudomonas* populations (Andersson et al., 2019). A classic example of this phenomenon is when a gene that confers resistance undergoes rapid changes in copy number due to the presence of repeated flanking regions, such as transposons (Andersson and Hughes, 2009; Papkou et al., 2020). Given that antibiotic resistance tends to be costly, an important consequence of HR is that resistance is lost from bacterial populations following antibiotics treatment, as we observed.

To directly test for HR, we sequenced 18 populations that were growing in medium containing colistin (Table 2). Sequencing revealed that 3/18 populations were *Micrococcus luteus*, implying that some contamination occurred during our experiments. *M*. *luteus* contamination was associated with a very conspicuous phenotype (yellow colonies) that was only detected very sporadically, allowing us to rule out the possibility that *M*. *luteus* contamination was common.

**Table 2:** Summary of variants detected in colistin-resistant populations

Variants	Functional role	Number of populations
pmrB (V28A)	two-component system sensor histidine kinase	4
pmrB (P254 S)	two-component system sensor histidine kinase	4
Amplification 1	putative lipoprotein	1
Amplification 2	type II secretion system protein J (Baldi et al., 2012) type II secretion system GspH family protein (Baldi et al., 2012)	1
none		5

Each sequenced population contained 0 or 1 variant, and the frequency of the detected SNPs was 100%, indicating a recent selective sweep.

Selective sweeps of mutations in the *pmrB* histidine kinase occurred in 8/15 populations, and this was the dominant molecular mechanism of HR. *pmrB* evolution was driven by repeated non-synonymous mutations at only two sites, revealing a high degree of parallel evolution. PmrAB has been shown to contribute to colistin HR through lipopolysaccharide (LPS) modification (Moskowitz et al., 2004), providing a simple explanation for the selective benefit of these mutations. Short amplifications of genes involved in LPS biosynthesis were found in a further two populations, providing further evidence that LPS alterations were the key mechanism of colistin HR in our experiments. Remarkably, the remaining 5/15 populations matched perfectly to the EP67 reference genome and did not contain any SNPs or gene amplifications.

### Estimating the mutation rate of pmrB

The high frequency of mutations in *pmrB* observed under colistin selection suggests that this gene may have an elevated mutation rate. We used the Luria-Delbrück method to estimate the rate of *pmrB* mutations (Lang, 2018; Luria and Delbruck, 1943). This simple method, which assumes that mutants are already present at the time of selection (in this case, treatment with colistin), estimates the mutation rate based on the cell titer and the frequency of cultures lacking mutants of interest. In this case, we assumed that the frequency of *pmrB* mutants in populations that recovered from colistin treatment was 8/15 across all of the 7 experiments (Table 1). Given this assumption, we estimate that *pmrB* mutations occurred at a rate of 2 × 10^-5^ per cell division (s.e. = 7.96 × 10^-6^; n = 7 assays).

### Conclusion

In this study, we explored responses to colistin treatment in many small populations of *P*. *aeruginosa*. Colistin treatment led to rapid cell death, and population size declined to approximately 10 cells after 8 h of colistin treatment (Figure 1). Remarkably, approximately 30% of populations recovered (Figure 1; Table 1) due to the slow growth of sub-populations of heteroresister cells with an unstable colistin-resistance phenotype (Figure 2). The dominant genetic mechanism of HR was mutations in PmrAB (Table 2), a two-component regulatory system that modifies LPS through the addition of aminoarabinose to lipid A (Moskowitz et al., 2004). HR was also driven by small amplifications of genes involved in LPS biosynthesis, providing further evidence for the importance of LPS alterations to colistin resistance. There are many known examples of HR to colistin (Andersson et al., 2019; Charretier et al., 2018; Cheong et al., 2019; Hjort et al., 2016; Karakonstantis and Saridakis, 2020; Lin et al., 2019), and the PmrAB system has been implicated in colistin resistance in many studies (Charretier et al., 2018; Cheong et al., 2019; Dosselmann et al., 2017; Jochumsen et al., 2016; Lee et al., 2016; Moskowitz et al., 2004). The key insight from our study is that the hypermutability of *pmrB* allows small *P*. *aeruginosa* populations to adapt to colistin treatment.

Stressful conditions, including antibiotics, are known to increase the mutation rate in bacteria (MacLean et al., 2013). Given this, it is possible that the mutagenic effects of colistin lead to mutations in *pmrB*. However, stress-induced mutagenesis creates mutations across the genome (Long et al., 2016), and the absence of second-site mutations provides good evidence that *pmrB* mutants were present at the time of colistin exposure due to the fact that this gene has an inherently high mutation rate. Our estimate of the mutation rate of *pmrB* is extraordinarily high (at least 2 × 10^-5^ per cell division) given that bacterial mutation rates are typically on the order of 10^-10^ per base pair per division (Schroeder et al., 2018) and that chromosomal resistance mutations typically arise at a rate of 10^-8^–10^-9^ per cell division (Andersson and Hughes, 2009; Krasovec et al., 2017).

Interestingly, mutations in the PmrAB system have been shown to facilitate airway colonization by *P*. *aeruginosa* and provide resistance to host antimicrobials that are produced as part of the innate immune responses, such as LL37 and lysozyme (Bricio-Moreno et al., 2018; Moskowitz et al., 2004). *P*. *aeruginosa* is an opportunistic pathogen, and our results raise the intriguing possibility that PmrAB may have evolved a high mutation rate to allow rapid adaptation to the host environment (Moxon et al., 1994) in a way that is analogous to the contingency loci that generate phase variation in *Haemophilus* and *Neisseria* (Bayliss et al., 2001; Moxon et al., 2006). Given the instability of the *pmrB* colistin-resistance phenotype, a key goal for future work will be to determine the extent to which *pmrB*-mediated HR drives treatment failure in patients undergoing colistin therapy for *P*. *aeruginosa* infections. Although *pmrB* mutations provide a simple mechanism for *P*. *aeruginosa* to adapt to colistin, *pmrB* mutations are known to have pleiotropic effects, including enhanced antibiotic susceptibility (Bricio-Moreno et al., 2018), that may effectively select against *pmrB in vivo*.

### Limitations of the study

Our study reveals a clear role for *pmrB* hypermutability in the evolutionary response of *P*. *aeruginosa* to colistin treatment. There are three main limitations associated with approach that we used. First, the molecular mechanism of *pmrB* hypermutability is unclear. Hypermutability in bacterial genomes is classically driven by repeated sequence motifs that are prone to acquiring insertions and deletions (Moxon et al., 2006), but repeats are conspicuously absent from *pmrB*, suggesting that alternative mechanisms drive the hypermutability of this gene (Horton et al., 2021). Second, our study did not provide insight into phenotypic mechanisms of adaptation to colistin. Approximately 1/3 of populations that recovered under colistin treatment did not contain mutations, and we can be confident that these represent true negatives given the depth of coverage of Illumina sequencing in these populations and the high quality of the EP67 reference genome that was used for read mapping. One possible explanation is that mutation-free populations reflect the growth of persister cells (Balaban et al., 2019). It is challenging to test this hypothesis, but it is difficult to see how populations of persister cells could continue to grow under colistin treatment (Figure 1C). An alternative explanation is that phenotypic plasticity contributes to colistin HR. According to this explanation, a small sub-set of *P*. *aeruginosa* cells are able to actively grow in the presence of colistin due to either inherent or induced expression of genes that modify the membrane to make it resistant to antimicrobial peptides like colistin (McPhee et al., 2003). Third, our study used the Luria-Delbrück method to estimate the mutation rate based on the distribution of detectable colonies. This method suffers from statistical limitations (Rosche and Foster, 2000; Stewart, 1994) and detection bias (Sun et al., 2018), and it is possible that we could have obtained a more accurate estimate of the *pmrB* mutation rate by sequencing alone. In spite of these limitations, our study provides clear evidence that the mutation rate of *pmrB* is orders of magnitude above the background mutation rate.

## STAR★Methods

### Key resources table

**Table:** 

REAGENT or RESOURCE	SOURCE	IDENTIFIER
**Bacterial and virus strains**		

Pseudomonas aeruginosa EP67	COMBACTE-Magnet Study	N/A

**Chemicals, peptides, and recombinant proteins**		

LB broth acc. Miller	VWR/Avantor	Cat# 84649.0500
Colistin (sulfate)	Cambridge Bioscience Ltd	Cat# 17584-5 g-CAY
LB BROTH WITH AGAR (MILLER)	Merck Life Science UK Limited	Cat# L3147-1KG
Glycerol	MP Biomedicals UK	Cat# 0215119491
Phosphate Buffered Saline	Merck Life Science UK Limited	Cat# P4417-50TAB

**Critical commercial assays**		

QuantiFluor® ONE dsDNA System	Promega	Cat# E4870
DNeasy Blood & Tissue Kit (250)	Qiagen	Cat# 69506

**Deposited data**		

Pseudomonas aeruginosa EP67	Wheatley et al., 2021	https://www.ncbi.nlm.nih.gov/biosample/16363815

**Software and algorithms**		

Trimmomatic version 0.39	Bolger et al., 2014	http://www.usadellab.org/cms/?page=trimmomatic
Breseq version 0.34.0	Barrick et al., 2014	https://barricklab.org/twiki/bin/view/Lab/ToolsBacterialGenomeResequencing
Bowtie2 version 2.3.5.1	Langmead and Salzberg, 2012	http://bowtie-bio.sourceforge.net/bowtie2/index.shtml
CNOGpro	Brynildsrud et al., 2015	https://cran.r-project.org/web/packages/CNOGpro/index.html
R version	The R Foundation for Statistical Computing	https://www.r-project.org/

**Other**		

Sequencing data	This paper	https://www.ncbi.nlm.nih.gov/bioproject Accession number: PRJNA809739, Table S1.xlsx
Counts of cultures showing growth under colistin treatment	This paper	Oxford University Research Archive at https://doi.org/10.5287/bodleian:kZ6VBbvky

### Resource availability

#### Lead contact

Further information and requests for resources and reagents should be directed to and will be fulfilled by the Lead Contact, Craig MacLean (craig.maclean@zoo.ox.ac.uk).

#### Materials availability

Strains and cell lines generated in this study are available upon request.

### Experimental model and subject details

#### Strain and culture conditions

In this study we used strain EP67, an MDR strain of *P*. *aeruginosa* ST17 that was isolated from an ICU patient with ventilator-associated pneumonia (Wheatley et al., 2021). The patient this strain was isolated from was treated with colistin, but this isolate was collected prior to colistin treatment. Unless otherwise stated, cultures were grown at 37°C in Luria-Bertani (LB) medium with shaking at 250 rpm or statically and solidified with 1% agar when appropriate. For all assays, colistin was freshly prepared from 5 mg/mL powder in water solution.

### Method details

#### Population dynamics experiment

Glycerol stocks of Strain EP67 were streaked out on LB plates and single isolated colonies were inoculated into 200μL of LB Miller and grown over 18–20 h at 37°C with shaking at 225 rpm. Overnight cultures were diluted in Phosphate Buffered Saline (PBS) and inoculated into 96 well polystyrene microtiter plates containing 200μL of fresh LB Miller supplemented with colistin at a concentration of 2 mg/L, such that the initial cell density was ≈1 × 10^6^ CFU/mL (i.e., 2 × 10^5^CFU/culture), as determined by serially diluting and plating out a sub-set of cultures on LB agar prior to incubation in colistin. Colistin cultures were then incubated at 37°C with shaking at 225 rpm. Samples of colistin cultures were serially diluted and spotted (5μL) on LB agar plates and CFUs were counted after overnight incubation at 37°C. The minimal dilution factor that was used was 10-fold, to ensure that colistin that was carried over to LB plates was at a sub-MIC dose (i.e., <0.2 mg/L). 100 independent replicates of this experiment were carried out across 4 different blocks. Viable cell density at times 0–8 h was estimated from the proportion of cultures that gave 0 CFUs at a dilution factor of 0.001 (time = 0) or 0.1 (all other time points) under the assumption that the number of CFUs/sample follows a Poisson distribution. Confidence intervals in the proportion of CFU-free samples were estimated from the normal approximation to the binomial distribution.

#### Population recovery experiment

Cultures of strain EP67 were pre-cultured and challenged with colistin using the same methods as in the population dynamics experiment above, except that a lower titre of cells was inoculated into colistin containing medium (≈5 × 10^4^ CFU/mL (i.e., 1 × 10^4^CFU/culture)). This experiment was replicated 933 times across 6 experimental blocks. Initial cell titre in each block was determined by serially diluting samples of 60 independent cultures in PBS, plating samples on LB, and counting CFUs after overnight incubation. Note that these samples were taken for CFU counts prior to incubation in colistin. All cultures were diluted 10 fold and 5 μL samples were spotted onto LB plates after 24 and 48 hr of incubation with colistin to quantify the frequency of population recovery.

#### Colistin resistance stability experiment

In this experiment, we challenged 171 independent cultures of EP67 with 2 mg/L colistin, as in the population dynamics experiment. After 24 h of incubation in colistin, each of these ‘selection cultures’ was diluted 10-fold into fresh LB medium, and these ‘recovery cultures’ were incubated overnight. Note that a 10-fold dilution was sufficient to reduce the concentration of colistin to a sub-MIC level (0.2 mg/L) that does not select for colistin resistance. After overnight incubation, recovery cultures were then passaged into (i)LB and (ii)LB + colistin (2 mg/L) by a 1000-fold serial dilution to set up ‘test cultures’ that were incubated overnight. After overnight incubation, all of the ‘recovery cultures’ and ‘test cultures’ were diluted 10-fold and 5 μL dots were plated on LB plates that were incubated overnight. Populations were counted as viable if they produced confluent colony growth, which corresponds to a culture density of at least 16,000 CFUs.

#### DNA extraction

DNA was extracted from 18 cultures that were growing in LB + colistin (2 mg/L) using QIAcube (QIAGEN) with DNeasy Blood and Tissue Kit (QIAGEN), and quantification of extracted DNA concentration was evaluated using QuantiFluor® ONE dsDNA System (Promega).

#### Sequencing and bioinformatic analysis

All samples were sequenced in the MiSeq illumina platform using the 300 × 2 Paired-end protocol (https://www.ncbi.nlm.nih.gov/bioproject/PRJNA809739, Table S1.xlsx). The sequencing experiment yielded a coverage of 64X-168X. Raw reads were quality controlled with Trimmomatic v. 0.39 (Bolger et al., 2014) using the ILLUMINACLIP (2:30:10) and SLIDING WINDOW (4:15) parameters. The coverage of the resulting quality-controlled reads ranged from 51X to 131X. Small variants were identified using breseq v. 0.34.0 (Barrick et al., 2014) in the polymorphism prediction mode, which implemented bowtie2 v. 2.3.5.1 (Langmead and Salzberg, 2012). Copy number variants were estimated with the default parameters of the package CNOGpro v. 1.1 (Brynildsrud et al., 2015) in *R* (R Development Core Team, 2010) using the *P*. *aeruginosa* reference genome EP67 (SAMN16363815, https://www.ncbi.nlm.nih.gov/biosample/16363815).

### Quantification and statistical analysis

#### Mutation rate estimation

The mutation rate at *pmrB* was estimated using the method of Luria and Delbruck (Luria and Delbruck, 1943). We estimated the proportion of cultures containing *pmrB* mutants by multiplying the number of cultures that tested positive for growth after 48hrs of incubation in colistin by the proportion of sequenced cultures containing *pmrB* mutations (8/15). The mutation rate at *pmrB* was estimated as -lnp (0)/N, where p (0) is the estimated proportion of cultures that did not contain a *pmrB* mutant and *N* is the initial number of CFU in the culture, as determined by counting CFUs from serial dilutions. The mutation rate was estimated for the initial population dynamics experiment and for the 6 blocks of the population recovery experiment. The error in the mutation rate was estimated as the standard error of the 7 independent estimates of the mutation rate.

## Data Availability

• The counts of cultures showing growth under colistin treatment data is available from the Oxford University Research Archive at https://doi.org/10.5287/bodleian:kZ6VBbvky. • Sequencing data is available from NCBI BioProject, Accession number:PRJNA809739; Table S1 xlsx. • This paper does not report original code. • Any additional information required to analyze the data reported in this paper is available from the lead contact upon request.

## Supplemental information

Table S1. Accession numbers for sequenced populations showing growth in colistin, related to Table 2
